# Reports on Hospitals of the United Kingdom

**Published:** 1913-12-20

**Authors:** Henry Burdett


### REPORTS ON
### Hospitals of the United Kingdom.

By SIR HENRY BURDETT, K.C.B., K.C.V.O.

SERIES III.

### LOWESTOFT HOSPITAL.

This is a hospital commenced in 1828, and now contains forty beds. It possesses the advantage of an ample site, well situated, and freely open to sunlight and air. The front elevation is rather attractive, thanks to the richness of its creepers and to the presence of an ornamental wooden verandah, where bedsteads containing patients can be placed, but not at right angles to the main building. These verandahs, like most parts of this hospital and its contents, date from a period anterior to the evolution of the modern hospital with its up-to-date appliances and equipment. The wards are of ample proportions, but the furniture, floors, bedding, and the appliances generally are, in several respects, mostly out of date. The lavatories and ward kitchens, where the latter exist, present a cruelly shabby appearance and the condition of the oil-cloth in some of them shows that the committee and the visitors to the hospital have sadly failed in their duty, or they never would have permitted these portions of the hospital to have fallen to their present condition of disrepair and worn-out makeshifts for the floors. The quality of some of the bedding, too, is sadly below the standard which ought to prevail.

### Starving the Hospital.

The operation theatre is well proportioned, but not too well lighted, and it contained so much that we may define as furniture that it presented rather the appearance of a surgical contractor's shop than that of a workshop for the surgeons, where important and difficult operations have frequently to be performed. The patients' clothes, linen, and other stores are placed at the head of the staircase on the first floor, where the whole of the vacant space is taken up by a pandemonium, pandemonium in the sense that here are associated together unventilated lockers, for the patients' clothes, for some portions of the surgical dressings, linen, and many other things. Some parts of this structure are backed with wood, whilst some of the lower portions back on to the wall, which gave manifest signs of being damp.

The conditions here represented, the evident starving of the wards and other portions of the hospital, from false notions of economy, including the defective bedding, which provides flock mattresses where none but horse hair should be used, must make this institution in its present condition a very depressing place for the sisters and nurses and for those members of the staff who have experience and knowledge of what a modern hospital ought to be and can be. Even to the patients of the better type the place must be depressing indeed. It was a wet day, it is true, when we visited this hospital, but bad as it was outside the lamentably low level of shabby meanness, to which things have been allowed to degenerate throughout the working portions of this little hospital were cruel facts to witness. They made one feel that those responsible could not really have ever been alive to their responsibilities, or they must long ago have decided either to close the institution, or to bestir themselves energetically to make it attractive in appearance, hygienically complete, and so a place to interest every inhabitant of Lowestoft and to secure his or her support and good will.

### What Lowestoft Hospital Needs Most!

What the institution needs is a man and a few women with driving force, to whom the best, and best alone, is good enough. A hospital cannot nowadays be run, and no attempt ought ever to be made to run it, in fulfilment of low standards. Experience teaches that a directorate where meanness, based upon a plea of no funds, has control, usually ends in a decrepit administration from which energy and any real sense of individual responsibility have long departed. The truth is, and it is better that we should state it quite bluntly, that if the administration of this hospital continues for a few more years to be worked under the present policy, resting as it does upon false economy without driving force, the institution will most certainly be closed.

We make this statement because we are fully aware of the changes which have been made in recent years in the hospitals of this country, and of others which are now imminent and must speedily occur. They involve the application of practical tests whereby the exact position and circumstances of a hospital, like that at Lowestoft, will be ascertained. They will demonstrate the quality of the administration and the possibility of its continuance from the actual condition of the institution when these tests have to be applied and a decision given. It is heartbreaking to come from the active hospital world of efficiency and progress to this neglected and stranded little hospital at Lowestoft.

### A Cruel Position and Inevitable Crisis.

The bright spots in the hospital are that good work is being done here by the medical staff, and that the nurses and sisters show a most commendable spirit and devotion in continuing to serve a hospital, where the Committee fail to support them as they ought to do, by providing up-to-date, thoroughly hygienic fittings and appliances with many essential things now absent from the wards and offices, where they ought to have had a place long ago. It is evident from the fact that Rufford's baths are to be found throughout the hospital, presenting as tempting and smart an appearance after twenty years' wear or more as they did when first put in, that the wisdom of obtaining the best of everything for hospital use is the truest economy. These baths were placed there, no doubt, at the instigation of those who loved the hospital and rightly felt that nothing could be too good for it. They stand out in testimony against those who, from nervous ineptitude, or for other reasons, have raised a cry of miscalled economy resting on false ideals, and so produced an absence of funds. There has been a lamentable falling away from a formerly high standard, with the result that the Lowestoft Hospital, from a structural point of view and so far as equipment and internal fittings are concerned, is to-day, a discredit to everyone who has sanctioned the continuance of its present lamentable condition. No one with a feeling of proper responsibility, or possessing a high sense of duty, could surely have refrained from lifting hand or voice to enforce reform, or in protest against the defects which are so patently noticeable by anyone with knowledge of what a hospital ought to be. Such defects would speedily disappear if this hospital had a body of managers who knew their duty and were determined to do it. Either the whole place should be taken in hand and brought up to date, or it should speedily disappear.

### A Courageous Practitioner.

Since the foregoing report was written we have received the account of the annual meeting of the hospital held on the 11th instant, published in the *Eastern Daily Press* of the 12th instant. This report is interesting for many reasons. First of all it shows that Dr. Worthington spoke out at the annual meeting and did good service to the institution by his insistence, that it was useless to appoint members on the committee, unless they attended and worked. A hospital is too important a responsibility for a real man to accept office in, unless he is prepared to give, and does actually give, all the time and energy which its best interests require from him. We are glad to see that Dr. Worthington, backed by Mr. J. A. Hewett, carried his point. The incident, however, has a special interest owing to an editorial note in the *Eastern Daily Press* of the 13th instant, in which the Editor states he entirely disagrees with one phrase we used in reporting on the Yarmouth Hospital. He quotes these words: "These Norfolk folk, when judged by other communities of equal size, do not as a whole contribute the sum they can properly give and ought to give to the hospital." We were thinking, as the Editor rightly assumes—may we apologise that the point is "clumsily expressed"—of Yarmouth and its hospital only. But the Editor must admit in all frankness that the spirit of our remark, at any rate, has a wider application than we had in mind when we wrote it, in view of the report of the Lowestoft meeting which inferentially condemns several members of the old committee. We have known Norfolk and greatly benefited in health from the happy years we once spent in this county. It always attracts us, and we are fully conscious, as we hope to show in due course, that Norfolk contains as generous, whole-hearted, and high-standard men and women as most places within the British Islands.

### Striking Facts from the Annual Meeting.

The Chairman, Colonel Cadge, gave some startling figures which fully confirmed the disastrous results consequent upon the past policy of the Committee, which we have criticised severely. He stated, for instance, that the annual subscriptions had decreased in eight years by £400, or something like 45 per cent. If that decrease is due, as the Chairman thought, "to deaths and removals," here surely is evidence of the most startling kind, that the hospital has sadly lacked driving force and energetic administration. We believe that the average standard of personal conduct for men and women in this country is high, and we cannot, therefore, agree with the Chairman that in a case like this "the future prospect of the hospital seems hopeless," though we do agree with him that unless the real men and women residents in Lowestoft wake up speedily, this hospital will "no doubt go into bankruptcy." It is encouraging to have the Chairman's authority for the statement that "Lowestoft is a growing town," and that "now especially a certain section of the inhabitants is increasing in wealth." Why is it, as the Chairman stated, that these prosperous people have so far failed to recognise their obligations to the Lowestoft Hospital? That is a matter for the new committee to lay hold of and sift to the bottom. We are glad to note that the fishermen seem to have set the whole town of Lowestoft an example to follow, for, whereas the amount received from congregational collections during last year was diminished by £59, the Committee were able to report "a very satisfactory increase in subscriptions and donations from the fishing industry."

### What Honour Demands from Lowestoft's Residents.

The hospital needs an increased income of £1,000 a year, and it requires another £1,000 at least to put the hospital in order and to equip it throughout. In the majority of the towns and cities of England the citizens regard it as an honour to work for the hospital, and to serve on its committee, and support it generously. How is it that this spirit, which was evidently that of the original supporters of the Lowestoft Hospital Committee years ago, has somehow been lost? Is it not time to search diligently and to recover that spirit? Is there not one man or woman, or several of them, who can move the whole intelligence of Lowestoft to turn to the Lowestoft Hospital, and never rest until they have put it into thorough order, and supplied it with the necessary sinews of war?

[We hope to publish next week the Report by Sir Henry Burdett on the Norfolk and Norwich Hospital.]

